# Association between Objectively Measured Sedentary Behaviour and Sleep Quality in Japanese Adults: A Population-Based Cross-Sectional Study

**DOI:** 10.3390/ijerph19053145

**Published:** 2022-03-07

**Authors:** Keita Kinoshita, Naoki Ozato, Tohru Yamaguchi, Motoki Sudo, Yukari Yamashiro, Kenta Mori, Yoshihisa Katsuragi, Takuji Yasukawa, Koichi Murashita, Shigeyuki Nakaji, Kazushige Ihara

**Affiliations:** 1 Department of Active Life Promotion Sciences, Graduate School of Medicine, Hirosaki University, Hirosaki City 036-8562, Japan; kinoshita.keita@kao.com (K.K.); oozato.naoki@kao.com (N.O.); mori.kenta@kao.com (K.M.); katsuragi.yoshihisa@kao.com (Y.K.); takuji.yasukawa@hirosaki-u.ac.jp (T.Y.); 2 Health & Wellness Products Research Laboratories, Kao Corporation, Tokyo 131-8501, Japan; yamaguchi.tohru@kao.com; 3 Department of Social Medicine, Graduate School of Medicine, Hirosaki University, Hirosaki City 036-8562, Japan; nakaji@hirosaki-u.ac.jp; 4 Personal Health Care Products Research Laboratories, Kao Corporation, Tokyo 131-8501, Japan; sudou.motoki@kao.com (M.S.); yamashiro.yukari@kao.com (Y.Y.); 5 COI Research Initiatives Organization, Graduate School of Medicine, Hirosaki University, Hirosaki City 036-8562, Japan; murasita@hirosaki-u.ac.jp

**Keywords:** physical activity, sedentary behaviour, sleep quality, intra-abdominal fat

## Abstract

The association between sedentary behaviour and sleep quality (SQ) remains unclear, partly due to the limited methodology for assessing sedentary time and the influence of obesity. This study aimed to examine the association between objectively measured sedentary time and poor SQ, as well as the association of visceral fat accumulation. This cross-sectional study used health check-up data obtained from 721 Japanese adults. Sedentary time and physical activity were measured using an accelerometer for ≥7 days, with ≥10 measurement hours per day. Poor SQ was determined by a Pittsburgh Sleep Quality Index score of ≥ 6. Visceral fat was measured using the abdominal bioimpedance method. A logistic regression model was used to analyse the association between sedentary time and SQ. We found that higher sedentary time was associated with poorer SQ. This association remained significant after adjustment for several covariates, including visceral fat. Compared with the lowest tertile of sedentary time, the second and highest tertile had a significantly higher OR of poor SQ (Tertile 2: OR = 2.06 [95% CI 1.14,3,73]; Tertile 3: OR = 2.76 [95% CI 1.49, 5.11]). These results suggest that managing sedentary time itself might contribute to improving SQ.

## 1. Introduction

Sleep problems constitute a global epidemic that threatens health and quality of life (QOL), with a prevalence of up to 56% worldwide [1,2,3]. Sleep quality (SQ), the subjective assessment of sleep, is associated with several health outcomes, such as brain health, cardiovascular health, metabolic health, and mortality [4]. A recent study in Japan demonstrated that poor SQ was observed in 16% of people over a wide age range and was associated with poor QOL [5]. Although hypnotic medications are widely used to promote good SQ, a free or low-cost method with a low risk of adverse side effects is needed for health promotion of the population.

Engaging in physical activity is a non-pharmacological method for increasing SQ. Greater amount of moderate-to-vigorous physical activity (MVPA) is associated with significantly better SQ and reduced frequency of sleep medication [6]. In 2020, the World Health Organization (WHO) guidelines on physical activity and sedentary behaviour recommended physical activity to improve sleep outcomes [7]. The guidelines also emphasised the association between sedentary behaviour and sleep; however, evidence for this was mainly focused on children and adolescents. Evidence for the relationship between sedentary behaviour and SQ in adults is inconsistent [8,9,10,11], partly due to the limited number of studies, differences in sample size, relatively narrow age range, differences in sedentary time according to race/ethnicity [12], physical activity level, and limited methodology. Additionally, most of these studies have assessed physical activity subjectively instead of using objective methods such as accelerometery, which is currently regarded as the gold standard [13]. Therefore, evidence is still needed regarding the association between objectively measured sedentary behaviour and SQ.

Sedentary behaviour is associated with obesity, and obesity is reportedly associated with sleep disturbance [14]. Previous studies have analysed the association between sedentary behaviour and SQ by adjusting body mass index (BMI), considering it as a modifier for the aforementioned association. Furthermore, the Asian population has a higher susceptibility to visceral fat accumulation, which is cause of metabolic syndrome [15], and visceral fat area (VFA) as one of the indexes of obesity is more strongly associated with sleep disturbance than BMI [16]. However, no studies have considered visceral fat to examine the association between sedentary behaviour and SQ, even in Asian populations.

Therefore, the aim of this study was to examine the association between objectively measured sedentary time and SQ, considering the association of VFA in Japanese adults.

## 2. Materials and Methods

### 2.1. Study Design and Participants

The Iwaki Health Promotion Project was launched in 2005. As part of the activities of the project, an annual health check-up was conducted for adults living in the Iwaki region of Hirosaki city, Aomori prefecture, located in northern Japan [17]. All adult residents (≈10,000) of this region were invited to participate based on resident registration, resulting in approximately 10% of the adult residents voluntarily participating. This population-based cross-sectional study analysed data obtained from the health check-up conducted from late May to early June in 2018 on 1056 individuals. This study was approved by the Ethics Committee of Hirosaki University School of Medicine (2018-012, 2018-063) and conducted in accordance with the principles of the Declaration of Helsinki. All study participants provided written informed consent. This study was registered with the University Hospital Medical Information Network (UMIN-CTR, https://www.umin.ac.jp, UMIN ID: UMIN000036741, accessed on 5 January 2022).

### 2.2. Measurements of Sedentary Time and Physical Activity

Sedentary time and physical activity were measured using an accelerometer (HW-100, Kao Corporation, Tokyo, Japan), which allowed 40 days of continuous recording at a sampling frequency of 64 Hz. The epoch length of the accelerometer was 4 s. The activity intensity was measured as previously described [18,19,20]. Briefly, accelerometer data were calculated as the time spent in each of the following three intensity levels: sedentary behaviour, ≤1.5 metabolic equivalent tasks (METs), and MVPA, ≥3 METs. A period of ≥35 min, where activity was not recorded using an accelerometer, was defined as the non-wear time. Sedentary time and MVPA were expressed as the mean daily hours across all adherent days (wear time ≥10  h/day) for all the participants.

The participants were instructed to wear the HW-100 on their waist throughout their awake period, while maintaining their daily activities, except during swimming or bathing. Additionally, the participants were instructed to begin wearing the HW-100 promptly after completing their health check-up and to return it after 10 days. The criterion for analysis was wearing the accelerometer for a total duration of ≥7 days (≥10 h/day) within the first 10 days after starting to wear the accelerometer.

### 2.3. Pittsburgh Sleep Quality Index (PSQI)

The Japanese version of the PSQI [21], validated using a control group among Japanese individuals with psychiatric disorders, was used to assess the SQ of the participants over the past month. The PSQI contains 19 self-rated questions aggregated into seven component scores (range of subscale scores, 0–3): sleep quality, sleep latency, sleep duration, habitual sleep efficiency, sleep disturbance, use of sleeping medication, and daytime dysfunction. The sum of these seven component scores yields a global score (range 0–21), which indicates subjective SQ. Higher PSQI global scores indicate poorer SQ. Poor SQ was defined as a PSQI global score of ≥6.

### 2.4. Visceral Fat Measurement

VFA was measured using a bioimpedance-type visceral fat meter (EW-FA90; Panasonic Corporation, Osaka, Japan), which is a certified medical device in Japan (No. 22500BZX00522000) for non-invasive VFA measurement [22]. Measurements obtained using this device are strongly correlated with those obtained using computed tomography [23], which is the gold standard for VFA measurement.

### 2.5. Other Variables

We obtained data regarding smoking habits using self-administered questionnaires prepared for the annual health check-up. Daily alcohol intake was determined using the Brief Diet History questionnaire [24,25]. Alcohol intake was categorised as none, low intake (<20 g/day), and high intake (≥20 g/day). Depressive status was assessed using the Centre for Epidemiologic Studies for Depression (CES-D) [26]. The questionnaire has been widely used to measure depressive symptoms in community populations and is also used as a screening tool for depression. Height, body weight, and BMI (calculated from height and body weight) were also measured.

### 2.6. Statistical Analysis

Given the strong correlation between accelerometer wear time and sedentary time (r = 0.72), we standardised sedentary time to 16 h/day of accelerometer wear time using the residuals obtained when regressing sedentary time on accelerometer wear time as previously described [27,28,29]. The participants were categorized into tertile groups based on the standardised sedentary time as follows: Tertile 1: sedentary time <  10.4 h; Tertile 2: 10.4 h  ≤  sedentary time  <  11.5 h; Tertile 3: sedentary time  ≥  11.5 h.

Participant characteristics are reported as median and interquartile range (IQR) or percentage. Continuous and categorical variables were compared between the two groups using Mann–Whitney U or Fisher’s exact tests and tertile groups using the Cochran-Armitage trend test.

To evaluate the association between sedentary time and the seven component scores of the PSQI, we used Spearman’s correlation coefficient. The method of Holm was applied to control the family-wise error rate in multiple comparisons. In addition, regression analysis with stepwise variable selection method was performed to investigate independent explanatory variables for the sedentary time.

Logistic regression analysis was used to determine the odds ratio (OR) and 95% confidence interval (CI) for SQ quality, which compared participants in the lowest tertiles of sedentary time to those in the higher tertile. We assessed linear trends across tertiles by including each participant’s tertile as an ordinal variable in the regression analysis. For the associations between VFA or BMI and poor sleep quality, the OR was represented per 1 standard deviation (SD) change in VFA or BMI.

Statistical tests were two-tailed, and statistical significance was set at *p*  <  0.05. As interactions by age and sex with sedentary behaviour were not statistically significant, pooled analyses were conducted. All analyses were performed using SPSS version 25 (SPSS Inc., Chicago, IL, USA) and the R environment (version 3.6.2; R Core Team, Vienna, Austria).

## 3. Results

In 2018, 1056 individuals participated in the health check-up. A total of 178 participants with incomplete clinical assessments, dietary data, PSQI data, CES-D data, or accelerometer data were excluded. Additionally, 157 participants who did not meet the inclusion criteria for the accelerometer data were also excluded. Finally, this study included 721 participants (277 men and 444 women) aged 20–88 years.

Among them, 13.5% reported PSQI ≥ 6. Table 1 shows the characteristics of the participants classified by their PSQI. Participants who were PSQI ≥ 6 were younger (*p* = 0.031), had higher CES-D scores (*p* < 0.001), higher sedentary time (*p* < 0.001), and were women (*p* = 0.052). Smoking status, alcohol intake, BMI, VFA, accelerometer wear time, and MVPA were not significantly different between the two groups.

**Figure 1:**
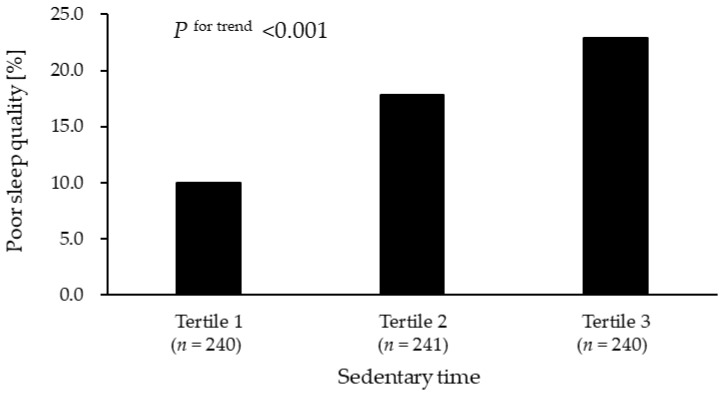
Associations of sedentary time with poor sleep quality. The value shown is the proportion of poor sleep quality (PSQI ≥ 6). Three groups according to the tertiles of sedentary time were assessed using Cochran–Armitage trend tests: Tertile 1, sedentary time <  10.4 h; Tertile 2, 10.4 h  ≤  sedentary time  <  11.5 h; and Tertile 3, sedentary time  ≥  11.5 h.

Figure 1 shows the proportion of PSQI ≥ 6 according to sedentary time tertiles. There was a significant association between sedentary time and PSQI ≥ 6 (*p* < 0.001); the proportion of PSQI ≥ 6 in Tertile 1 was 10.0%, Tertile 2 was 17.8%, and Tertile 3 was 22.9%.

Table 2 shows the correlation coefficients between sedentary time and the seven PSQI component scores. After the Holm adjustment, higher sedentary time was found to be significantly associated with worse sleep latency score (r = 0.11, *p* = 0.003) and worse daytime dysfunction score (r = 0.13, *p* = 0.021). The regression analyses (Table S1) indicated that daytime dysfunction was the most significantly associated component with sedentary time

Table 3 shows the OR (95% CI) for PSQI ≥ 6 according to the tertiles of sedentary time. After adjustment for several covariates (Model 2), there was a significant association between sedentary time and PSQI ≥ 6 (P for trend < 0.001); compared with Tertile 1, Tertile 2 and Tertile 3 had significantly higher ORs for PSQI ≥ 6 (Tertile 2: OR = 2.07 [95% CI 1.15, 3.75]; Tertile 3: OR = 2.98 [95% CI 1.62, 5.48]).

Table S2 shows the association between PSQI ≥ 6 and VFA and BMI, respectively. After adjustment for several covariates, there was a significant association between VFA (1SD) and PSQI ≥ 6 (Model 2: OR = 1.31 [95% CI 1.03, 1.67]). There were no significant associations between BMI (1SD) and PSQI ≥ 6 (Model 2: OR = 1.21 [95% CI 0.98, 1.50]).

**Figure 2:**
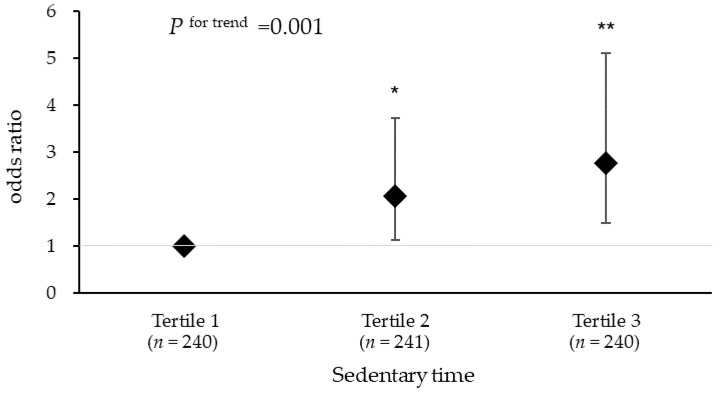
Odds ratio for the association between sedentary time and PSQI ≥ 6. Data are presented as odds ratios and 95% confidence intervals (error bar). Logistic regression models were used by adjusting for age, sex, smoking status, alcohol intake, CES-D score, MVPA, and VFA. Tertile 1, sedentary time <  10.4 h; Tertile 2, 10.4 h  ≤  sedentary time  <  11.5 h; Tertile 3, sedentary time  ≥  11.5 h. * *p* < 0.05; ** *p* < 0.01.

Figure 2 shows the results of the association between sedentary time and PSQI ≥ 6 and further adjustment for VFA in Model 2. There was still a significant association between sedentary time and PSQI ≥ 6 (P for trend = 0.001); compared with Tertile 1 (<10.4 h/day), Tertile 2 and Tertile 3 had significantly higher ORs for PSQI ≥ 6 (Tertile 2: OR = 2.06 [95% CI 1.14,3,73]; Tertile 3: OR = 2.76 [95% CI 1.49, 5.11]).

## 4. Discussion

To the best of our knowledge, this is the first study to investigate the association between objectively measured sedentary time and SQ while considering the association of visceral fat. Recent studies have reported that prolonged sedentary time and poor SQ were significantly associated with higher visceral fat [30,31], indicating that the association between sedentary time and SQ may be partially influenced by visceral fat. We found that a higher sedentary time was independently associated with poorer SQ after adjustment for VFA. These results suggest that reducing sedentary time itself might contribute to better SQ.

We assessed sedentary behaviour, as well as MVPA, using an objective method, and a significant association was found between sedentary time and SQ even after adjustment for MVPA. A number of studies have examined the association between physical activity and sleep outcomes. Recent WHO guidelines recommended regular physical activity to improve sleep outcomes, such as SQ, and provided evidence for the association between sedentary behaviour and sleep outcomes; however, it was mainly focused on children and adolescents, especially for the association between sedentary time and sleep duration [7]. Although several studies have examined the association between sedentary behaviour and SQ in adults, the results have been inconsistent [8,9,10,11]. This is partly attributed to the differences in methodology used to measure sedentary behaviour among the studies.

In contrast to our results, Sloan et al. reported that there was no independent association between objectively measured sedentary time and SQ in adults in Singapore [11]. This can be partly explained by the differences of the study population in sedentary time. The Japanese population spends more time in sedentary behaviour compared to the populations of other countries [12]. The sedentary time duration in the present study was approximately 10.9 h/day, whereas in Sloan’s study, it was 7.86 h/day. Although between-study comparisons of physical activity levels are impeded by methodological differences in accelerometer measurement, the association of sedentary time with SQ might differ by countries depending on the differences in the amount of time spent on engaging in sedentary behaviour. The longer sedentary time in Japanese enabled us to consider the effect of much longer sedentary time on the SQ, which was not considered in Sloan’s study.

In our study, we found significant correlations between sedentary time and two components of PSQI: latency and daytime dysfunction. The physiological mechanisms between sedentary behaviour and SQ are not well understood, and previous studies have suggested a potential mechanism between exercise and SQ. Exercise can decrease sleep latency and increase slow-wave sleep [32,33], which may partially explain the association between sedentary time and sleep latency. As for daytime dysfunction, a bidirectional association might be considered. Some studies reported that poor SQ was associated with low scores on the Short Form-36 questionnaire, which assessed the quantity and quality of daily physical activity [5,34]. Further studies are needed to reveal how sedentary behaviour affects SQ, independent of physical activity.

Previous studies have reported that obesity and metabolic disorders are associated with poor SQ [35,36]. Although BMI is frequently used in clinical settings to assess obesity status (general obesity), recent studies have shown that VFA (index of abdominal obesity) is more strongly associated with hypertension, type 2 diabetes, dyslipidaemia, and cardiovascular disease than BMI [37,38,39]. In addition, VFA is more strongly associated with sleep disturbance than BMI [16]. We found that VFA was significantly associated with SQ, whereas BMI was not, which may be attributed to the aforementioned difference between VFA and BMI. However, Dekker et al. reported that VFA was significantly higher in individuals with poor SQ, but the association was attenuated after adjustment for total body fat [40]. This is partly explained by the fact that Asian individuals have a higher susceptibility to metabolic syndrome, with visceral fat accumulation even in individuals with a lower BMI, compared with Western individuals [15]. In addition to reducing sedentary behaviour, a population-specific approach for coping with obesity may be required for better SQ. Conversely, another possible explanation could be that poor SQ can be linked to increasing risk for obesity via hormonal regulations, such as leptin [31]. Thus, further longitudinal studies are needed for elucidating the direction of these associations.

We found a significant linear association between longer sedentary time and poor SQ. As an individual’s activity time in a given day is finite, reducing sedentary time leads to an increase in other physical activities. Although physical activity is reported to be good for health, recent reviews have suggested that higher levels of occupational physical activity are associated with an increased risk of poor SQ [41]. Therefore, it is necessary to not only reduce sedentary time but also consider how to increase other physical activities. To achieve good SQ, non-occupational activities such as exercise, walking, and activities of daily living would be beneficial for reducing sedentary time.

The strengths of this study include measurement of VFA using an abdominal bio-impedance method, and objective measurement of sedentary time and physical activity using an accelerometer. However, this study had some limitations. First, the cross-sectional design of our study could not establish a causal relationship between sedentary behaviour and SQ. Edwards et al. reported that an experimental increase in sedentary behaviour decreased SQ, which supports our causal hypothesis [42]. Second, the loss of participants due to insufficient accelerometer data could have led to selection bias. Third, there might have been an overestimation of the sedentary time, since sedentary behaviour was defined based on intensity levels (≤1.5 METs) using an accelerometer which cannot distinguish between sitting and standing postures. Fourth, although we adjusted for several covariates, there might have been residual confounding variables. The wide age range in this study may bias the results of the association between sedentary behaviour and SQ. Finally, as this study was confined to participants from a particular country, region, race, and limited sample size, reproducibility should be confirmed by the inclusion of a large number of participants from different regions and/or races.

## 5. Conclusions

Objectively measured sedentary time was significantly associated with poor SQ, especially for daytime dysfunction. Furthermore, although VFA was significantly associated with poor SQ, higher sedentary time was independently associated with poor SQ after adjustment for VFA. Reducing sedentary time itself might help to improve the SQ of people with high sedentarism. Further longitudinal or interventional studies are required to confirm the effectiveness of reducing sedentary time on SQ.

## Figures and Tables

**Table 1:** Participant characteristics classified by PSQI global score.

	PSQI < 6 (*n* = 599)	PSQI ≥ 6 (*n* = 122)	*p*-Value
Age, years	58 (44–66)	52 (39–65)	0.031
Sex, % women	59.9	69.7	0.052
Smoking Status, %			
Never	62.9	68.0	0.105
Former	23.9	15.6	
Current	13.2	16.4	
Alcohol intake, %			
None	43.1	51.6	0.239
Low (<20 g/day)	34.6	29.5	
High (≥20 g/day)	22.4	18.9	
CES-D score	5 (2–10)	12 (6–20)	<0.001
BMI, kg/m^2^	22.5 (20.3–24.7)	22.8 (20.6–25.3)	0.789
VFA, cm^2^	75 (48–108)	79 (50–111)	0.774
Accelerometer wear time, h/day	15.5 (14.2–16.6)	15.6 (14.4–17.2)	0.341
Sedentary time, h/day ^1^	10.9 (9.87–11.8)	11.4 (10.6–12.2)	<0.001
MVPA, h/day	0.38 (0.26–0.53)	0.39 (0.27–0.53)	0.616
PSQI global score	3 (2–4)	7 (6–8)	<0.001

Abbreviations: BMI, body mass index; VFA, visceral fat area; MVPA, moderate–vigorous physical activity; PSQI, the Pittsburgh Sleep Quality Index. Data are shown as median (IQR) or percentage. Mann–Whitney U tests were used for continuous variables, and Fisher’s exact tests were used for categorical variables. ^1^ Sedentary time was expressed as the estimated hours of sedentary time per day, given as standardised 16 h of accelerometer wear time.

**Table 2:** Correlation between sedentary time and the seven component scores of PSQI.

	PSQI Global Score	Sleep Quality	Sleep Latency	Sleep Duration	Habitual Sleep Efficiency	Sleep Disturbance	Use of Sleeping Medication	Daytime Dysfunction
Sedentary time	0.10 *	0.06	0.11 *	−0.01	0.03	0.04	0.06	0.13 **

Values show Spearman’s correlation coefficients between sedentary time and PSQI global and seven component scores. * Holm adjusted *p* < 0.05, ** Holm adjusted *p* < 0.01.

**Table 3:** Odds ratio of PSQI ≥ 6 according to tertiles of sedentary time.

	Tertiles of Sedentary Time			
	Tertile 1 (*n* = 240)	Tertile 2 (*n* = 241)	Tertile 3 (*n* = 240)	*p*-Value for Trend
Model 1	1.00 (reference)	1.87 (1.09, 3.20)	2.44 (1.44, 4.14)	<0.001
Model 2	1.00 (reference)	2.07 (1.15, 3.75)	2.98 (1.62, 5.48)	<0.001

Values shown are odds ratios (95% confidence intervals). Logistic regression models were used in the present study. Model 1 was adjusted for age and sex. Model 2 was adjusted for Model 1 plus smoking status, alcohol intake, CES-D score, and MVPA. Tertile 1, sedentary time <  10.4 h; Tertile 2, 10.4 h  ≤  sedentary time  <  11.5 h; Tertile 3, sedentary time  ≥  11.5 h.

## Data Availability

The data presented in this study are available on request from the Hirosaki University COI Program Institutional Data Access/Ethics Committee (contact via e-mail: coi@hirosaki-u.ac.jp) for researchers who meet the criteria for access to the data. Researchers must be approved by the research ethics review board at the organisations of their affiliations. The data cannot be shared publicly because of ethical concerns.

## Supplementary Materials

The following is available online at https://www.mdpi.com/article/10.3390/ijerph19053145/s1, Table S1: Association between the seven component scores of PSQI and sedentary time. Table S2: Association between PSQI ≥ 6 and VFA and BMI, respectively.
