# Supplementary material for: Association between Objectively Measured Sedentary Behaviour and Sleep Quality in Japanese Adults: A Population-Based Cross-Sectional Study
Source: Int J Environ Res Public Health. 2022 Mar 7;19(5):3145. doi: 10.3390/ijerph19053145 (PMC8910757; doi:10.3390/ijerph19053145)
Supplement: Supplementary file 1 [file ijerph-19-03145-s001.zip › ijerph-1584468-supplementary.pdf]

Table S1. Association between the seven component scores of PSQI and sedentary time.

	**B**	**β**	**95% CI**	***p*-Value**
daytime dysfunction	0.31	0.14	(0.15, 0.47)	<0.001
sleep latency	0.20	0.10	(0.06, 0.34)	0.005

Stepwise linear regression models were used. β = standardized regression coefficient. CI: Confidence interval.

Table S2. Association between PSQI ≥ 6 and VFA and BMI, respectively

	**VFA**	***p*-Value**	**BMI**	***p*-Value**
Model 1	1.26 (1.01, 1.57)	0.043	1.15 (0.95, 1.40)	0.143
Model 2	1.31 (1.03, 1.70)	0.026	1.21 (0.98, 1.50)	0.070

VFA, visceral fat area; BMI, body mass index. Values shown are odds ratios (95% confidence intervals) for per 1SD change in VFA or BMI. Logistic regression models were used in the present study. Model 1 was adjusted for age and sex. Model 2 was adjusted for Model 1 plus smoking status, alcohol intake, CES-D score, and MVPA.
